# Attitudes of final-year medical students towards psychiatry as a specialisation at a university in South Africa

**DOI:** 10.4102/sajpsychiatry.v32i0.2660

**Published:** 2026-09-16

**Authors:** Muhammed Tayob

**Affiliations:** 1 Department of Psychiatry, School of Clinical Medicine, Faculty of Health Sciences, University of the Witwatersrand, Johannesburg, South Africa

**Keywords:** attitudes, perceptions, psychiatry, specialise, stigmatisation, students

## Abstract

**Background:**

Despite mental illness being a major burden in South Africa, there is a shortage of psychiatrists compared to global rates. By understanding final-year medical students’ attitudes towards psychiatry, new recruitment strategies can be implemented to overcome this hurdle.

**Aim:**

This study aimed to assess final-year medical students’ attitudes toward psychiatry and to identify demographic and experiential factors that influence the intention to specialise in psychiatry.

**Setting:**

The University of the Witwatersrand.

**Methods:**

A cross-sectional, quantitative survey was conducted among final-year medical students between July 2024 and October 2024 using the Mental Illness: Clinicians’ Attitudes (MICA-2) Scale.

**Results:**

Among 171 students, the overall mean MICA-2 score was 40.43 (standard deviation = 8.16), indicating moderately positive attitudes. Females demonstrated significantly more favourable attitudes (mean = 39.35) than males (mean = 43.08, *p* = 0.022). Older students (> 33 years, *n* = 8, 4.7%) had slightly more positive attitudes than younger age groups, but these findings were not significant (*p* = 0.268). Exposure to a psychiatry clinical rotation did not positively influence students’ attitudes (*p* = 0.257), and personal fulfilment and work-life balance were the predominant deciding factors with regard to specialisation choice.

**Conclusion:**

There is no significant stigmatisation towards psychiatry across all cohorts. The study instead reflects differing levels of positive attitudes towards psychiatry. However, this did not translate to students pursuing psychiatry as a specialty, as they feel it does not offer personal fulfilment and work-life balance.

**Contribution:**

Future strategies must focus on work-life balance and personal fulfilment when attempting to increase the recruitment rate into psychiatry.

## Introduction

Psychiatric disorders are a major contributor to global disease burden.^1,2,3^ This is via direct (such as affecting individuals by reducing their ability to work, study or interact with others) and indirect (caregiver burden and strain on those around the patient) means.^4,5,6^ As a result, there is a reduction in the productivity of a nation and a greater load on the healthcare services of that nation.^4,5,6^

The above emphasises the importance of treating mental health in South Africa and, as such, the need to train more psychiatric specialists to be able to treat these patients. Psychiatry remains an unpopular specialisation choice, especially in low- to-middle income countries (LMICs) like South Africa.^7^ In South Africa, there is only a psychiatrist-to-population ratio of 0.31 per 100 000, well below the global median of 1.273.^8^ This underscores that there is a dearth in the number of psychiatrists needed to adequately treat the mental healthcare users (MHCUs) in South Africa.

Two recent studies in the United Kingdom (UK) found that factors promoting recruitment into psychiatry include personal interest, demographics, past life experiences and the potential for achieving work-life balance.^7,9^ Potential deterrents include the profession’s perceived lower status, stigmatisation, inadequate exposure during training and the emotional demands of the work.^7,9^

While in medical school, students are already considering their career choices, and the exposure they receive in each of their rotations will likely impact their decisions.^10,11,12^ If they have a negative attitude towards psychiatry, they may be less likely to specialise in that field.^10,11^

A 2011 multinational cross-sectional survey showed that female students were more inclined than male students (21% vs 16%, *p* = 0.007) towards psychiatry.^10^ However, interest in the field waned later in their training, because of experiences as a student and poor working conditions. Similar results were seen in a UK multi-year study.^13^

A Nigerian study showed that stigma towards psychiatry, limited clinical exposure and lack of role models led to less interest in specialising in psychiatry.^11^

The recent study by Ochse and Lowton found that 97.2% of fourth-year medical students had a low prevalence of stigmatising or negative attitudes toward psychiatry.^14^ African students showed slightly less interest in psychiatry compared to other racial groups. This is concerning in a country whose largest population demographic is African.^14^

Understanding the attitude of medical students towards psychiatry as a specialised field is important, particularly in South Africa where the number of psychiatrists is notably lower than in other medical specialisations.^8^

A possible way to entice students towards psychiatry is to emphasise the ability to offer comprehensive care to patients who are marginalised by society and to tackle thought-provoking cases.^10^

The significance of this research in South Africa is emphasised by the distinctive cultural, socioeconomic and healthcare system circumstances that could impact the perspectives of medical students regarding pursuing a specialisation in psychiatry.^15^ Although international studies offer valuable insights, it is important to consider that differences in contextual elements between where the study is conducted and other parts of the world may limit the applicability when translating the findings. Even within South Africa, there are vast contextual differences between provinces; thus, a study done in one may not accurately reflect the attitudes of students in another.

The existing literature underscores the psychiatrist shortage in the country and highlights the diverse factors that influence the choice of specialisation. While some of these factors appear universal, others are context-specific, underscoring the need for a study tailored to South Africa. Therefore, this study intends to better understand the attitude that final-year medical students have towards psychiatry and the factors that influence their attitude, their desire to specialise, and more specifically, their desire to specialise in psychiatry.

### Aim and objectives

The aim of the study was to examine the attitudes of final-year medical students towards psychiatry as a specialisation. The objective was to describe the relationship between that attitude and the students’ demographics, exposure to psychiatry and desire to specialise in psychiatry.

## Research methods and design

### Study design

A quantitative cross-sectional design was adopted in this study. A descriptive approach was employed to show the frequency and distribution of measured attitudes.

### Setting

The study was conducted at the University of the Witwatersrand (WITS). The study occurred between July 2024 – October 2024. The choice of the institution was solely based on the researcher’s association with the institution as a student.

### Study population and sampling strategy

The population consisted of all final-year medical students at WITS, who numbered 338. This university also allows postgraduate students to register to study medicine, meaning that there is a wide range of ages among possible participants. Part of their training was to undergo a 6-week rotation in psychiatry at any of the hospitals associated with WITS. The researcher estimated the necessary sample size to represent the population of 338 students with a 95% confidence level and a 5% margin of error using Equation 1:

$$n=\frac{N}{\left[1+N(e{)}^{2}\right]}$$ [Eqn 1]

In this formula, *n* is the sample size, *N* is the population size, and e is the margin of error. By setting *N* to 338 and e to 0.05, the arithmetic was as follows:

*n* = 338 / [1 + 338 × (0.05)^2^]

*n* = 338 / [1 + 338 × 0.0025]

*n* = 338 / [1 + 0.845]

*n* = 338 / 1.845

*n* ≈ 183

To meet the 95% confidence level and 5% margin of error criteria, a total of 183 surveys were deemed necessary.

### Data collection

The research employed the Mental Illness: Clinicians’ Attitudes (MICA-2) scale, which has been specifically designed to measure the attitudes of clinicians and students towards psychiatry.^16^ Permission to use the tool was granted by the Thornicroft Foundation.

The MICA-2 scale has been validated through rigorous testing and has been used in multiple settings.^14,16^ It comprises 16 statements rated on a six-point Likert scale (from ‘strongly agree’ to ‘strongly disagree’).

The possible scores range from a minimum of 16 to a maximum of 96, with a median of 56.^16^ Lower total scores on the MICA-2 generally indicate more favourable attitudes and lower stigma, whereas higher scores reflect more negative or stigmatising views.^16^

All final-year medical students at WITS, who were willing and able to provide informed consent for participation in the study, were included.

As a result of the timing of data collection, it was anticipated that only some participants would have already completed their psychiatry rotation at the time of the survey. This natural difference was then used to compare attitudes in these two groups based on clinical exposure.

Students were approached on campus grounds and requested to participate in the study. If they agreed, a link was provided to them that would allow completion of the questionnaire. As most final-year medical students were seldom on campus grounds, as they were busy with clinical duties, participants were asked to recruit other students who were in their rotation, thereby improving the response rate (snowball sampling).

The data were captured using the REDCap (Vanderbilt University, Nashville, Tennessee, United States) software. The first section was the informed consent section that the participant had to complete before continuing with the rest of the questionnaire. Section two asked for demographic information, including gender, age, race and whether the student had completed the mandatory 6-week clinical rotation in psychiatry. With regard to race, the official population categories in South Africa are black, white, Indian, coloured and other. This study split the last category of other into Asian and mixed race. The third section employed the MICA-2 scale. The final section asked questions directly relating to the factors affecting the participant’s desire to specialise and their opinion of psychiatry specifically.

### Data analysis

Before the data could be analysed, it needed to be checked to ensure that it would be appropriate for the study.

The total number of participants who initiated the survey and gave consent to participate (completed section one) between July 2024 and October 2024 was 239. Of those, 15 did not complete the demographics, 26 did not complete the MICA-2 scale, and five did not complete section C. These participants were removed as the response was invalid; thus, there were only 193 responses remaining.

Subsequently, it was noted during data cleaning that some participants had noted their ages below that theoretically possible for being in the final year of medical school. If we assume that the earliest a person can start the 6-year medical degree is 17 years old, turning 18 years old that same year, then the lowest possible age to be in the final year would be 22 years, turning 23 years old that same year. Thus, the 22 responses that indicated the participant’s age was below 22 were also removed, as the validity of these responses could not be verified to come from actual final-year medical students. This left only 171 responses (Figure 1).

**FIGURE 1:**
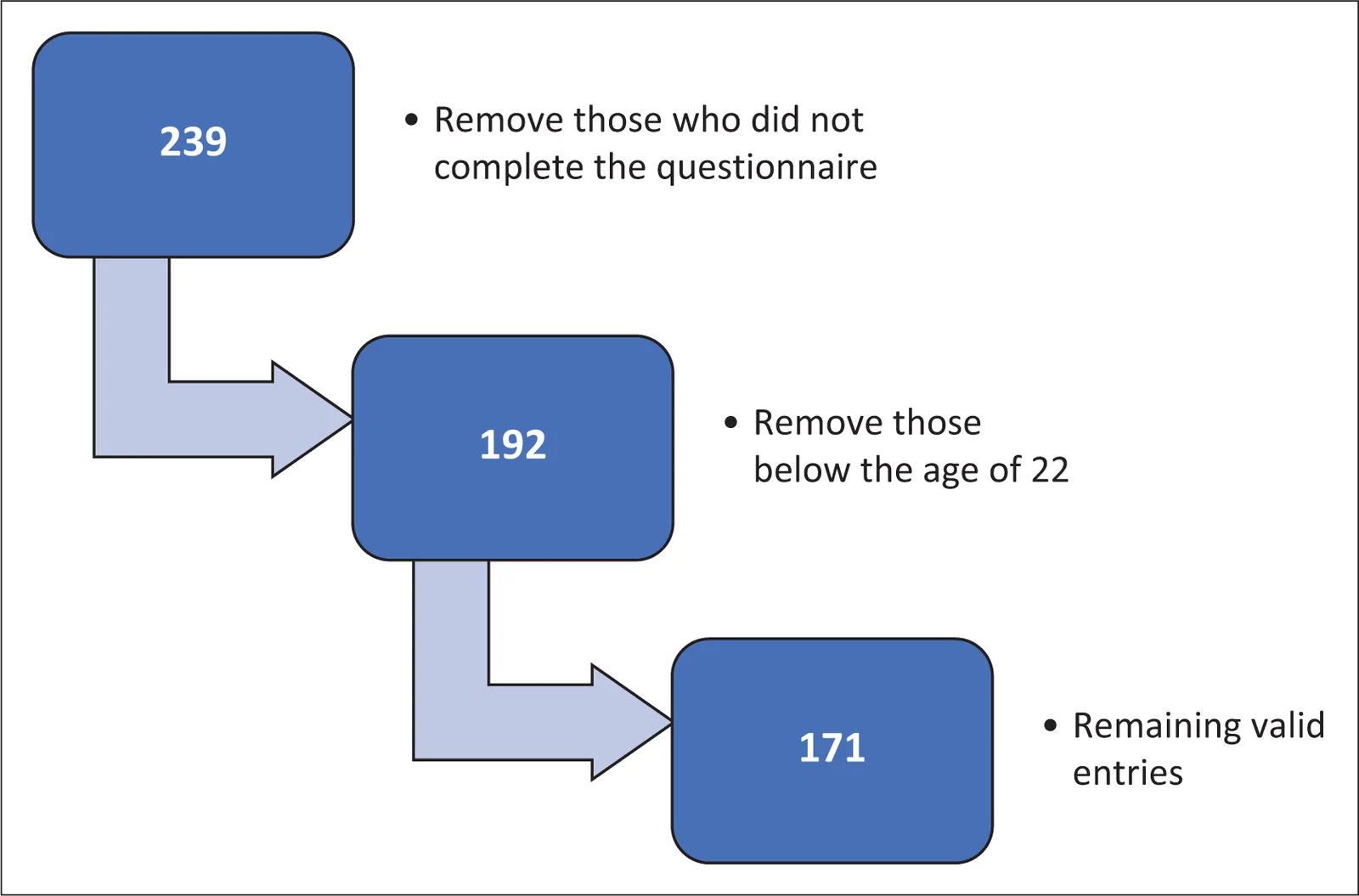
Data cleaning process.

This is 12 below the required number of participants needed to meet the confidence interval of 95% (183). This is because the study was halted once the required number of participants was obtained (183), and only during data cleaning were some participants removed; thus, the total qualified responses are below the required level.

The reason why the difference (12) in required participants is not accounted for directly in any of the above numbers is that some of the participants removed for being underage would have also been removed in subsequent data cleaning stages, thus skewing the sample size. If the age construct were overlooked, the total number of participants would have remained at 183. This is because the study was closed once 183 fully completed responses were obtained.

The dataset was exported into a statistical software package (SPSS version 26) for analysis. Descriptive measures, such as frequencies, means and standard deviations (SD), were first calculated to map the overall distribution of variables.

Cross-tabulations were used to explore whether demographic variables, including completion of a psychiatry rotation, correlated with attitudes. Chi-square tests were performed to test associations between categorical variables, while *t*-tests (or nonparametric alternatives) helped compare mean attitudes where relevant. Multiple logistic regression was carried out to detect which factors best predicted a positive inclination toward psychiatry as a future specialty. A *p*-value below 0.05 was used to define statistical significance.

### Ethical considerations

Ethical clearance to conduct this study was obtained from the WITS Human Research Ethics Committee (Medical) (Ref. No. M240511 M240628-A-0002). Before beginning the study, the participants had to read and agree to an online informed consent form that was part of the questionnaire. All participants were informed of their right to withdraw from the study at any point. The questionnaire did not request names or any form of personal information. The participants were informed that there were no incentives for participating in the study and what to do in the case that the study caused any distress. The participants were previously unknown to the investigator, and there was no relationship between them; thus, no conflicts of interest could arise.

Data were kept on the REDCap online service where only the principal investigator had access to it, ensuring that the data remained secure.

## Results

Table 1 breaks down the MICA-2 results first by demographics, then by the responses for two points in the questionnaire.

**TABLE 1:** Summarised results according to demographic sub-divisions.

Category	Subgroup	Total (*n*)	%	Mean MICA 2 score	± SD	IQR
Total sample		171	100.0	40.43	8.16	11.0
Gender	Male	50	29.2	43.08	8.16	9.8
	Female	119	69.6	39.35	7.97	12.0
	Other	2	1.2	38.00	7.07	5.0
Race or ethnicity	Asian people	6	3.5	39.33	6.15	9.2
	Black people	41	24.0	38.39	6.77	9.0
	Coloured people	8	4.7	38.38	9.61	10.2
	Indian people	54	31.6	42.31	8.59	11.8
	Mixed-race people	5	2.9	39.40	8.85	15.0
	White people	57	33.3	40.60	8.47	11.0
Age (years)	22–27	141	82.5	40.30	8.13	10.0
	28–33	22	12.9	42.41	8.06	9.2
	> 33	8	4.7	37.12	8.53	14.2
Completion of psychiatry rotation	Yes	130	76.0	40.80	8.39	11.0
	No	41	24.0	39.24	7.34	11.0
Desire to specialise in psychiatry	Yes	55	32.2	37.09	7.70	8.5
	No	116	67.8	42.01	7.92	11.0
A high-paying salary is a deciding factor for me on which field to specialise in	Agree	92	53.8	41.46	8.02	12.0
	Disagree	79	46.2	39.23	8.20	11.0

SD, standard deviation; MICA, mental illness: clinicians’ attitudes; IQR, interquartile range.

Findings for the entire sample population showed a mean score of 40.43 (SD = 8.16), indicating moderately positive attitudes. The median score 39.0, which falls well below the threshold of 56 (anything above a score of 56 is associated with higher levels of stigma).^16^

Sub-dividing the participants shows that most were female, accounting for 69.6% (119 participants), followed by 29.2% male (50 participants) and a small proportion identifying as ‘Other’ (1.2%, 2 participants).

Female participants demonstrated a mean score of 39.35 with a median of 38.0, indicating generally more positive views, compared to males with a mean of 43.08 and a median of 42.0 (*p*-value = 0.022).

The interquartile range (IQR) for females is relatively narrow: 12.0. The minimum and maximum scores for females range from 25.0 to 62.0, showing that while some participants hold more stigmatising views, the majority fall below the threshold for high stigma.

In contrast, male participants had an IQR of 9.8, which is lower than that of females. Male scores range from a minimum of 26.0 to a maximum of 63.0, with a more substantial portion of male respondents exhibiting more stigmatising attitudes.

For participants identifying as ‘Other’, the mean score was 38.0 with a median of 38.0 (both scored the same), comparable to the male and female distributions, but given the small sample size (*n* = 2), conclusions about this group are limited. Comparisons between participants identifying as ‘Other’ and both male and female groups, however, show no statistically significant differences in attitudes

The ethnicity of the group showed a racial spread: white people (33.3%), Indian people (31.6%), black people (24.0%), coloured people (4.7%), Asian people (3.5%) and mixed-race people (2.9%).

A one-way analyses of variance (ANOVA) to compare MICA-2 scores across different racial groups gave a *p*-value of 0.026, which is statistically significant at the 0.05 level. This suggests that there are meaningful differences in MICA-2 scores between at least some of the racial groups. However, ANOVA only tells us that a difference exists, not where it lies.

A Tukey Honestly Significant Difference (HSD) post-hoc test to identify where the differences lie between racial groups’ MICA-2 scores showed that the only statistically significant difference is between Indian and black students (Indian students had higher MICA-2 scores, i.e., more stigmatising attitudes; mean difference = 3.92 points, *p* = 0.039). All other racial or ethnic group comparisons were non-significant.

Figure 2 graphically represents the different age cohorts and their respective MICA-2 scores.

**FIGURE 2:**
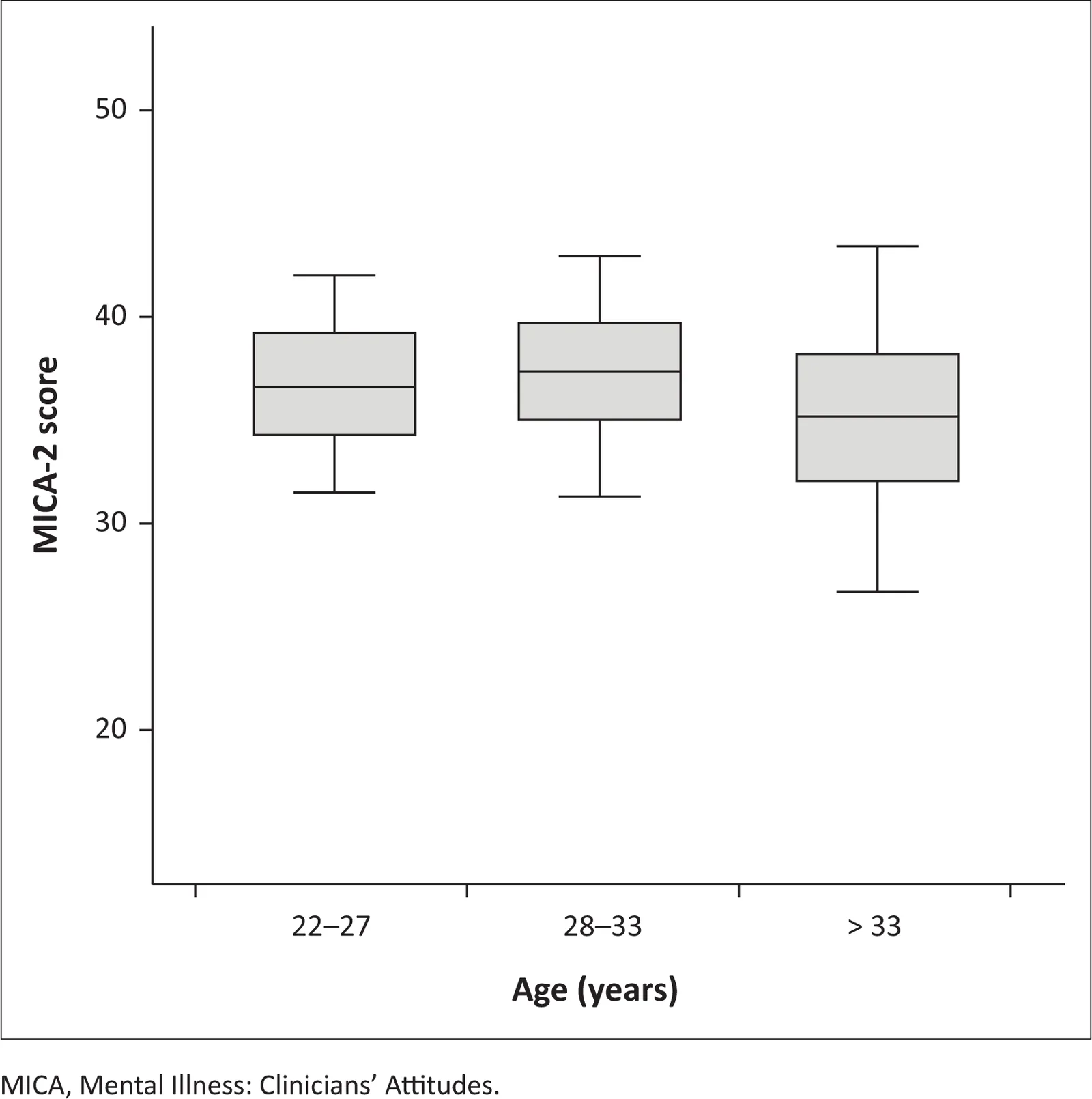
Box-and-whisker plot of Mental Illness: Clinicians’ Attitudes-2 scores by age cohort.

The ages of the participants were split into cohorts: 22–27 years (82.5%), 28–33 (12.9%) and >33 (8%). The age group of 22–27 years had a mean score of 40.30, whereas the age group of 28–33 years had a mean score of 42.41, and the age group > 33 years had a mean of 37.12 (*p*-value of 0.268), suggesting that attitudes between the age groups are not statistically significant.

Males were more likely than females to agree that a high-paying salary is a deciding factor when choosing a specialty (66.0% vs 48.7%), but the difference does not reach conventional statistical significance (*p* = 0.098).

Among the population, 90% agreed that quality of life is a critical factor in their specialisation choice (*p* < 0.001). Participants who prioritise quality of life when choosing a specialisation are more likely to have a low MICA-2 score (*p* = 0.031).

By contrast, there is a strong monotonic relationship between the importance of a high salary and the MICA-2 score. Students who strongly agreed that high salary drives their specialty choice had the highest mean MICA-2 score: 44.65, while students who strongly disagreed that salary matters had the lowest mean MICA-2 score: 37.38 (*p* = 0.030). This could indicate that students feel that psychiatry is not a high-paying field.

Table 2 compares the response to the statement ‘I wish to specialise in psychiatry’ between those who have completed the 6-week clinical rotation in psychiatry and those who have not.

**TABLE 2:** Cross-tabulation of ‘I wish to specialise in psychiatry’ with ‘Completed the 6-week clinical rotation of psychiatry’.

Completed rotation?	Definitely or probably interested (1–3)		Neutral (4)		Probably or definitely not interested (5–6)	
	*n*	%	*n*	%	*n*	%
Yes	32	25.2	17	13.4	78	61.4
No	18	40.0	7	15.6	20	44.4

**Total**	**50**	**29.1**	**24**	**14.0**	**98**	**57.0**

There is a large disparity in this study between those who had not completed the rotation (24.0%) and those who had completed the psychiatry rotation (76.0%). There was no statistically significant difference between the mean MICA-2 scores of both groups: 39.24 versus 40.80 (*p* = 0.257).

When looking at the effects of the 6-week clinical rotation on the desire to specialise in psychiatry, participants who had completed the psychiatry rotation were less likely to show interest in the field (*p* = 0.082).

Students who had already firmly decided on their specialty were much more likely to attribute that decision to their medical-school clinical exposure (48.4% said ‘definitely or probably yes’ vs. only 20.3% among those who had not yet decided). Students who were still undecided were far more likely to say clinical exposure did not play a major role (31.6% definitely or probably no vs. only 12.9% among decided students) (*p* = 0.38).

Students who believed that psychiatrists are necessary to the functioning of the healthcare sector had a mean MICA-2 score of 38.63, whereas students who were ambivalent or disagreed had a mean MICA-2 score of ≥ 50.9 (*p* < 0.001).

## Discussion

### Key findings

This study was aimed at examining attitudes toward psychiatry among final-year medical students at a South African university and to identify factors that influence the choice or rejection of psychiatry as a career path. The findings of the study suggest that final-year medical students have moderately positive attitudes towards psychiatry. Literature suggests that those with stigmatising views are less likely to specialise in psychiatry.^16^

The findings of this study showed that men have the highest MICA-2 score on average (43.08), but this is still below the threshold of stigmatisation (considered a level of 56). This is in keeping with the findings of Ochse and Lowton,^14^ who found low stigmatising levels among all the cohorts in their study.

There were, however, key differences between cohorts, suggesting that female participants generally maintain more positive attitudes towards psychiatry than males, with male participants showing more variability and a higher prevalence of stigmatising views

However, the unequal distribution of males versus females in respondents may have impacted this result. This difference in gender-based scores could stem from cultural expectations, or an attitude of psychiatry as a field that values discussion and empathy, both of which are found to be common among females.^10,11,12^

Examining the data, younger participants (22–27 years) show low MICA-2 scores, suggesting more positive attitudes towards psychiatry. This trend shifts in the 28–33 years age group, where scores increase, reflecting less favourable attitudes towards psychiatry.

### Discussion of key findings

Positive attitudes were correlated with a desire to specialise in psychiatry. While financial factors are relevant, they are not as decisive as lifestyle considerations. Those who had completed the 6-week rotation had similar attitudes compared to those who had not, suggesting that exposure alone does not necessarily shift opinion. This contrasts with the previous studies that showed that having a good mentor and role model in psychiatric training led to better attitudes towards psychiatry.^11,12^ However, this study did not assess the quality of the psychiatry rotations that the students experienced, only whether they had gone through one or not.

A total of 32.2% of participants indicated some level of desire to specialise in psychiatry. This is noteworthy given the vast array of options available for specialisation for medical officers (this already excludes the plethora of options besides specialising).

An overwhelming majority believed that mental health is critical to every individual’s wellbeing, but much fewer believed that psychiatrists are essential to the healthcare sector. This disparity could explain why, although psychiatry is respected, it is not widely pursued by students.^13,14^

The analysis of MICA-2 scores across different age groups reveals a relationship between age and attitudes toward psychiatry. This indicates that attitudes toward mental illness vary across age categories, which may reflect evolving perspectives shaped by clinical experience, professional expectations or societal attitudes encountered over time.

This difference could reflect less exposure to entrenched societal attitudes toward mental health that may influence older participants. This finding indicates that the >33 age group holds attitudes that are less stigmatising, perhaps reflecting the cumulative influence of age and professional experience.

The mean values when adjusted for race suggest that, despite some minor variations, there is no notable disparity in the levels of stigma or acceptance among the racial groups. The overall similarity across racial groups may reflect the influence of shared educational experiences within the cohort, potentially fostering a consistent outlook on mental health and psychiatry.

### Strengths and limitations

A key strength of this study lies in its contextual relevance. By focusing on final-year medical students at a South African university, the research accounts for the effects that training and exposure to psychiatry in an academic setting may have on a student’s attitude towards psychiatry. The use of a validated instrument (MICA-2) enhances the reliability and comparability of the findings. Furthermore, the analysis incorporated multiple demographic and attitudinal variables, allowing for a nuanced understanding of how gender, age, race and clinical exposure interact with attitudes towards psychiatry.

However, several limitations must be acknowledged. Firstly, the sample size (*n* = 171) did not meet the calculated requirement for statistical power, thus possibly not presenting a fair reflection of the study population. Secondly, the gender distribution was skewed, with female participants overrepresented (69.6%), which could have influenced overall findings, particularly since women displayed more favourable attitudes. As such, they would be more willing to participate in studies about psychiatry. This would be participation bias, and true conclusions about the attitudes of the males in the class cannot be drawn as a result. Further research may focus on this group that did not participate to provide more insights.

Thirdly, the cross-sectional design captures attitudes at a single point in time. As a result, it cannot determine causality or track changes over time. Moreover, although the MICA-2 is a validated tool, it may not fully capture culturally nuanced or subconscious biases present in the South African context.

Fourthly, the findings are limited to one institution and may not be generalisable to all medical schools in South Africa, particularly those with different student demographics or curricular structures. Future studies may benefit from a longitudinal design, a more balanced demographic representation and multi-site sampling to deepen understanding and enhance generalisability.

### Implications of recommendations

The author of this research suggests that further research be done on the cohorts that were under-represented in this study, particularly males, to ensure more reliable data in future studies. The information gathered suggests that quality of life for students precedes their need for financial gain. Having fewer stigmatising attitudes towards psychiatry improves desire to specialise in that field, therefore, programmes to reduce stigma should be instituted by the university to overcome that hurdle.

## Conclusion

There is no significant stigmatisation towards psychiatry across all cohorts. The study instead reflects differing levels of positive attitudes towards psychiatry. Those in the female cohort have a significantly more positive attitude. This study highlights that while final-year medical students generally hold positive attitudes towards psychiatry and acknowledge its critical role within the healthcare system, this is not necessarily associated with a strong desire to pursue it as a career. Students appear to prioritise factors such as personal fulfilment and work-life balance over purely financial incentives when considering specialisation options.

These findings point to a possible shift in the attitude towards psychiatry, when compared to earlier research, and a valuable opportunity for continued medical education and workforce policy to maintain this shift. By improving clinical exposure, offering robust mentorship and actively reshaping the narrative around psychiatry’s career potential, including its lifestyle benefits and intellectual rewards, it may be possible to inspire more students to consider this vital and underserved field.
